# Investigation on the Energy-Absorbing Properties of Bionic Spider Web Structure

**DOI:** 10.3390/biomimetics8070537

**Published:** 2023-11-10

**Authors:** Baocheng Xie, Xilong Wu, Xuhui Ji

**Affiliations:** 1 Key Laboratory of Advanced Manufacturing Intelligent Technology of Ministry of Education, Harbin University of Science and Technology, Harbin 150080, China; 2 School of New Energy Engineering, Weifang Institute of Technology, Qingzhou 262500, China

**Keywords:** spider web, energy-absorbing properties, structure parameters, regression equation

## Abstract

In recent years, spider webs have received significant attention due to their exceptional mechanical properties, including strength, toughness, elasticity, and robustness. Among these spider webs, the orb web is a prevalent type. An orb web’s main framework consists of radial and spiral threads, with elastic and sticky threads used to capture prey. This paper proposes a bionic orb web model to investigate the energy-absorbing properties of a bionic spider web structure. The model considers structural parameters such as radial line length, radial line cross-sectional diameter, number of spiral lines, spiral spacing, and spiral cross-sectional diameter. These parameters are evaluated to assess the energy absorption capability of the bionic spider web structure. Simulation results reveal that the impact of the radial line length and spiral cross-sectional diameter on the energy absorption of the spider web is more significant compared to the radial line cross-sectional diameter, the number of spiral lines, and spiral spacing. Specifically, within a radial line length range of 60–80 mm, the total absorbed energy of a spider web is inversely proportional to the radial line length of the web. Moreover, the number of spiral lines and spiral spacing of the spider web, when within the range of 6–10 turns and 4–5.5 mm, respectively, are proportional to the total energy absorbed. A regression equation is derived to predict the optimal combination of structural parameters for maximum energy absorption. The optimal parameters are determined as follows: radial line length of 63.48 mm, radial line cross-sectional diameter of 0.46 mm, ten spiral lines, spiral spacing of 5.39 mm, and spiral cross-sectional diameter of 0.48 mm.

## 1. Introduction

The mechanical properties of biological structures have attracted great interest. Among the numerous energy-absorbing structures, spider webs have stood out for their high strength and energy-absorbing performance [1,2,3]. The primary function of spider webs is to withstand strong impact forces and absorb the kinetic energy brought by prey, preventing its escape. This indicates that spider webs have excellent elasticity and toughness. Therefore, studying spider silk and designing the structure of spider webs has become a possibility. Spider silk is the most common bionic material in nature, with properties that are far superior to most artificial materials [4]. Spider silk is a high-performance bionic material with characteristics such as being lightweight and high-strength, with tensile strength even higher than steel but with strong elasticity as well [5]. The mechanical properties of different types of spider silk are primarily determined by their different protein compositions [6,7]. Compared with other artificial nylon materials, spider silk materials can improve their strength without harming their fracture toughness. In addition to the excellent properties of spider silk materials, the spider web structure is a unique combination of geometry and mechanics, serving as an efficient energy-absorbing structure. It can quickly absorb the impact brought by insects and severe vibrations [8,9,10]. To better visualize the structural composition of spider webs, a method for building spider web models has been developed, resulting in a complete model of the spider web structure [11]. It is worth noting that the characteristics of spider silk have been fully studied [12,13,14]. However, current scholars focus more on the material properties of spider webs than their structure.

This study primarily investigated the energy absorption behavior of spider web structures subjected to large-scale vibrations. Research on the energy absorption performance of such structures could be well applied in areas such as wearable devices or aerospace engineering. A three-dimensional model of the spider web structure was designed using Solidworks, and the energy absorption characteristics of the spider web structure were analyzed using Ansys. A parametric study examined the effects of different parameters, including radial length, radial cross-sectional diameter, number of spiral lines, spacing between spiral lines, and cross-sectional diameter of spiral lines.

## 2. Mathematical Model of the Orb Web

### 2.1. Analysis of Spider Silk Deformation Energy Absorption

**Figure 1:**
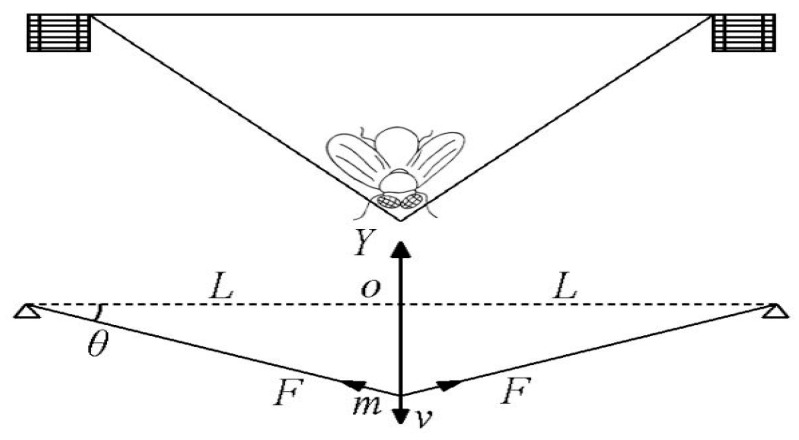
Spider silk impact resistance model.

The effect of spider web energy absorption depends on the mechanical properties of the spider web. Therefore, the mechanical model of the spider web under impact loading is first studied. Suppose a spider web is subjected to an insect of mass m, which impacts the midpoint of the spider web with a velocity v. As the ends of the spider web are held in place, the stickiness of the silk causes the insects to stick to the web and vibrate with it. The spider web is deformed to consume the kinetic energy of the insect’s impact. This is shown in Figure 1. Therefore, to simplify the model, the insect can be equated to a mass of m that impacts the midpoint of the spider silk with an initial velocity and oscillates with the spider web.

A geometric analysis of Figure 1 shows that:

(1) $$y=\frac{L}{2}\tan\theta$$

where y is the position of the mass in the vertical direction; θ is the angle between the spider silk and the horizontal line. The increase in the length of the spider silk is:

(2) $$\Delta =\frac{L}{\cos\theta }-L$$

The strain of the spider silk is:

(3) $$\varepsilon =\frac{\Delta }{L}=\frac{1}{\cos\theta }-1$$

According to Gosline et al. [15], the mechanical analysis of the radial line of the round web spider shows that the expression of the stress-strain relationship of the radial line is as follows:

(4) $$\left\{\begin{array}{c} Loading\to \sigma =\left\{\begin{array}{l} 10\varepsilon \\ 0.2+3.6\left(\varepsilon -\varepsilon_{y}\right)\\ 0 \end{array}\right.\begin{array}{c} 0<\varepsilon \leq 0.02\\ 0.02<\varepsilon \leq 0.27\\ \varepsilon >0.27 \end{array}\\ Uninstallation\to \frac{d\sigma }{d\varepsilon }=10 \end{array}\right.$$

where σ is the material stress; ε is the material strain; and $\varepsilon_{y}$ is the material yield strain. The stress-strain relationship of the helix can be expressed by the polynomial power function:

(5) $$\begin{array}{cc} \sigma =a\left(\varepsilon +b\varepsilon^{n}\right) & 0<\varepsilon <2.7 \end{array}$$

where a and b are constants. Thus, during loading or unloading, the stress and strain of the spider silk are always linearly related, so the stress σ of the spider silk can be uniformly expressed as:

(6) $$\sigma =G\frac{1}{\cos\theta }+\left(H-G\right)$$

where G and H are constants determined by different loading conditions.

Therefore, the theoretical solution of the dynamic response of the spider silk subjected to the shock depends on the initial conditions, and the dynamic response of the spider silk is up-and-down oscillation.

### 2.2. Number of Diameter Lines and Number of Spiral Coils Study

The number of radial lines and spiral lines are the most important features of the spider web structure, and the combination of different numbers of radial lines and spiral lines determines the strength of the spider web [16].

A reasonable relationship between the number of radial lines and the number of spiral lines can be deduced from the number of insects hitting the spider web glandular filaments simultaneously. When an insect with mass m and velocity v impacts a spider’s web, the web undergoes deformation and absorbs energy due to the force exerted by the insect. When the deformation reaches a critical point, the spider’s web experiences maximum force, which we designate as F. The maximum attachment force possessed by a single spider spiral wire filament is f, then, the number of spiral lines φ to which the insect is attached simultaneously is the ratio of F to f.

If the radius of the outermost spider spiral is R, the cross-sectional area of insects is r. To ensure that insects do not pass through the spacing of two adjacent spirals, the number of spiral lines *n* is:

(7) $$n=\frac{F}{f}\cdot \frac{R}{2r}$$

If $f_{0}$ is the maximum bearing force of a single diameter wire, the number of insects of radius r hitting the spider silk diameter wire μ is the ratio of F to $f_{0}$.The number of radial lines (*Y*) is:

(8) $$Y=\frac{2\pi R}{2r}\cdot \mu =\frac{\pi RF}{rf_{0}}$$

Therefore, the number of radial and spiral lines is directly influenced by the spider web’s maximum area and the insects’ full impact force. An increase in the spider web area and insect impact force leads to an increase in the number of radial lines, thus enhancing the strength of the spider web. Additionally, increasing the number of spiral lines helps disperse impact energy into the radial lines more effectively. Hence, the load-bearing capacity of radial lines and the adhesion force of spiral lines directly impact the number of radial and spiral lines. In the simulation model, we can alter the cross-sectional area of radial and spiral lines to establish the relationship between different ratios of radial lines and the number of spiral lines regarding the absorption of impact energy in spider webs.

## 3. Simulation Analysis of Energy Absorption of Bionic Spider Web

In this study, based on the fact that round web spider webs show excellent mechanical properties in capturing insects, we constructed a bionic spider web model designed as a combination of radial and spiral lines. The radial lines are the main structural lines radiating outward from the center of the spider web. In contrast, the spiral lines are the capture lines of the spider web, which are distributed circumferentially from the center outward. In the process of the spider web being impacted by insects, due to the structural specialties of the spider web, the spider web distributes the force to each radial line, mitigates the impact, and absorbs the energy through the up and down oscillations. The length of the trails, the number of helical coils, and the spacing of the cobwebs significantly affect the energy absorption.

### 3.1. Finite Element Modeling

#### 3.1.1. Simulation Model Construction and Boundary Condition Setting

**Figure 2:**
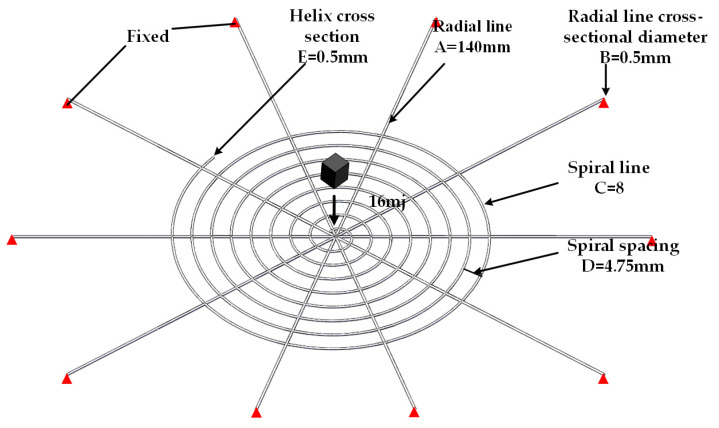
Insects hitting the spider web model.

A 3D model of the spider web was constructed using SolidWorks, as shown in Figure 2, to simulate the spider web’s dynamic response when subjected to impact loads. The length of the radial lines (A) is 140 mm, the diameter of the radial line cross-section (B) is 0.5 mm, the number of spiral lines (C) is 8, the spiral spacing (D) is 4.75 mm, and the spiral cross-sectional diameter (E) is 0.5 mm. The constructed spider web model was imported into ANSYS LS-DYNA, and the ends of the radial lines were fixed using boundary constraints. Based on the parameter settings for the spider web impact by previous researchers, to simulate this process, a mass of 260 mg with an initial velocity of 12 m/s is used in the finite element method to impact the nodes in the web. The initial total kinetic energy of the block is 16 mJ. To explore the optimal energy absorption effect of biomimetic spider webs, since the central point of the spider web has the best energy absorption effect, we set the impact location as the main node of the spider web. Due to computational limitations and considering mesh sensitivity, the mesh size was set to 0.02 mm. Moreover, based on the spiral surface characteristics of the spider web, a hexahedral mesh was generated using a sweeping method.

#### 3.1.2. Parameter Settings of the Simulation Model

In this simulation, the resin material UTR9000 is used instead of spider silk. The resin material has excellent formability and higher tensile and compressive strengths, and its material parameters are easy to obtain. Subsequent research will continue to use resin materials to explore the coherence of biomimetic printing structures. Moreover, resin materials’ mechanical properties, elastic behavior, and fracture characteristics have been extensively studied, making them suitable for simulation studies. We employed an isotropic elasticity theory model in the simulation setting and set the material damping value to 0.2. The parameters of the resin material are detailed in Table 1.

### 3.2. Dynamic Response Process of Energy Absorption of Spider Web

#### 3.2.1. Deformation Analysis for Spider Web Simulation

**Figure 3:**
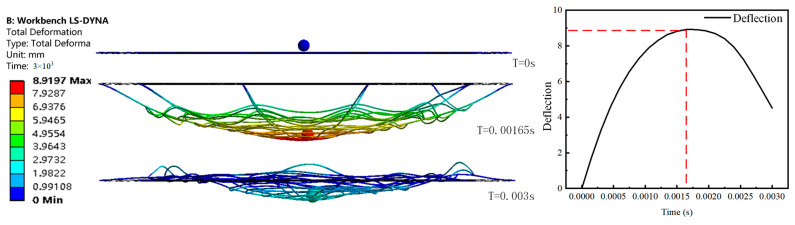
Spider web transformation diagram.

From Figure 3, it can be observed that during the impact of the object on the spider web, at time 0.00163 s, the spider web experiences maximum deformation. The spider web’s radial lines are stretched, while the spiral lines exhibit oscillations during the impact process. As the maximum deformation concludes, the spider web begins to rebound. This is due to the fact that the spider web material and structure store a significant amount of elastic potential energy during the impact, which is released as the deformation of the spider web reduces.

#### 3.2.2. Analysis of Energy Absorption in Spider Web Simulation

**Figure 4:**
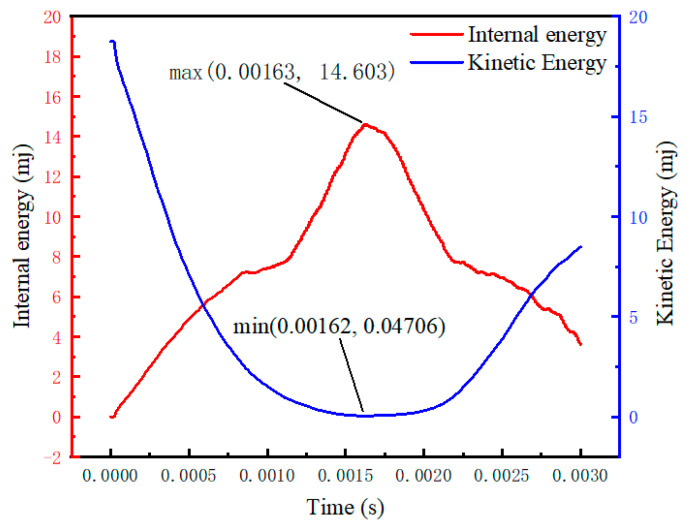
Cobweb deformation energy absorption.

From Figure 4, it can be observed that during the impact of the object on the spider web, the deformation of the spider web increases. The object’s kinetic energy is converted into the elastic potential energy of the spider web deformation, resulting in a decrease in kinetic energy and an increase in the internal energy of the spider web. At time t = 0.00163 s, the spider web absorbs the maximum energy, which is 14.603 mJ, while the object’s kinetic energy is minimized at 0.04706 mJ. It leads to the release of deformation in the spider web, resulting in a decrease in the internal energy of the spider web and an increase in the object’s kinetic energy.

### 3.3. Spider Web Energy Absorption Effect Evaluation Index

Total energy absorption EA refers to the amount of kinetic energy that the spider web converts into its own internal energy after blocking the impact of flying insects. The higher the value, the better the energy absorption effect.

(9) EA=f(x0)+g(x1)

where f(x0) represents the total absorbed energy of the radial wire and g(x1) represents the total absorbed energy of the spiral lines.

### 3.4. Simulation Test Data Analysis Based on the Response Surface Method

The response surface method improves the efficiency of the experiment and can visualize the influence law of the spider web parameters. In this paper, a second-order model is used to approximate the optimal solution to improve accuracy, and the model is shown in Equation (10).

(10) $$y=\beta_{0}+\sum_{i=1}^{m}\beta_{i}x_{i}+\sum_{i=1}^{m}\beta_{ii}x_{i}{}^{2}+\sum_{i<j}^{m}\beta_{ij}x_{i}x_{j}+\varepsilon$$

where $\beta_{i}$ is the linear effect of $x_{i}$; $\beta_{ii}$ is the second-order effect of $x_{i}$; and $\beta_{ij}$ is the interaction effect of $x_{i}$ and $x_{j}$.

#### 3.4.1. Simulation Design and Results of the Effect of Cobweb Structure Parameters on Energy Absorption

In this analysis, the Box-Behnken Design (BBD) method was utilized to investigate the impact of spider silk structure parameters on energy absorption. A design plan was established with five variables as factors, namely the radial line length (A), radial line cross-sectional diameter (B), number of spiral turns (C), spacing between coils (D), and spiral cross-sectional diameter (E). The variables and their levels were set as follows in Table 2.

Based on the levels of the variables above, a simulation analysis was designed with a total of 41 sets of different structural parameters for the spider web, considering the total energy absorption (EA) as response indicators. Simulation data obtained from the analysis are presented in Table 3. The five variables, namely the radial line length (A), radial line cross-sectional diameter (B), number of spiral lines (C), spiral spacing (D), and spiral cross-sectional diameter (E), were considered factors, each with three levels, resulting in a 5-factor, 3-level, 1-response design.

In order to verify the significance level of this model and each factor and to ensure the credibility of the obtained law about the effect of spider web structure parameters on the energy absorption effect, the simulation was tested for significance. The significance test is a screening for the chance of the null hypothesis holding. The invalid hypothesis is established when the opportunity of the weak belief being based reaches 5%.

#### 3.4.2. Total Energy Absorption Significance Analysis

The significant data on the influence of spider web radial line length (A), radial line cross-sectional diameter (B), number of spiral lines (C), spiral spacing (D), and spiral cross-sectional diameter (E) on total energy absorption (EA) are summarized in Table 4.

#### 3.4.3. Establishing Regression Equations with a Normal Probability Distribution of Residuals

From the table, it is possible to see that the three factors of spider web radial line length (A), number of spiral lines (C), and spiral spacing (D) have significant effects on the total absorption energy (EA) of spider web. In contrast, the impact of the radial line cross-sectional diameter (B) and spiral cross-sectional diameter (E) on the total absorption energy (EA) is significantly insignificant. According to the idea of the BBD design method and simulation data, the regression fit of the response as a function of relevant factors was performed as follows:

(11) $$\begin{array}{ll} EA= & 14.05-0.525A+0.035B+0.127C+0.286D-0.071E-\\ & 0.03AB-0.115AC-0.1245AD+0.022AE-0.135BC-\\ & 0.051BD-0.08BE-0.075CD+0.037CE-0.14DE+\\ & 0.19A^{2}+0.052B^{2}+0.268C^{2}+0.13D^{2}+0.06E^{2} \end{array}$$

**Figure 5:**
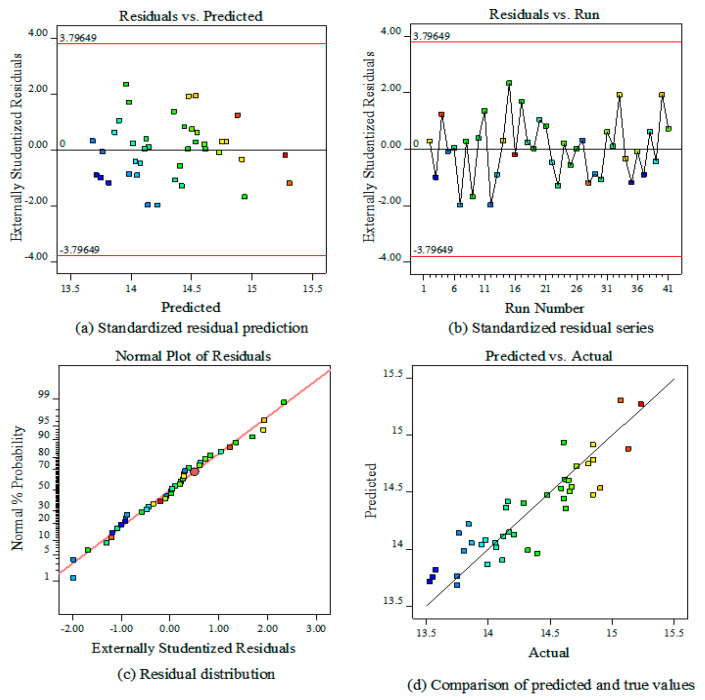
(**a**) Standardized residual prediction. (**b**) Standardized residual series. (**c**) Residual distribution. (**d**) Comparison of predicted and true values.

Figure 5a shows the relationship between spider webs’ predicted total adsorption energy and the variation of internal standardized residuals. The data points are randomly scattered on both sides, indicating that the simulated data model is meaningful in practice. Figure 5b shows the residual series of the total energy absorption test data on the spider web, and the data points alternate within the error range, indicating that the model is more reliable and credible. Figure 5c shows the model’s internal standardized residual distribution plot, where the residuals are roughly distributed along a diagonal line, indicating a good fit for the model. Figure 5d shows the comparison between the predicted and actual values of the model for the total energy absorption of the spider web, and the relevant data points are evenly distributed in the joint measurement of the straight line, indicating that the model is of good quality.

#### 3.4.4. Response Surface Analysis

**Figure 6:**
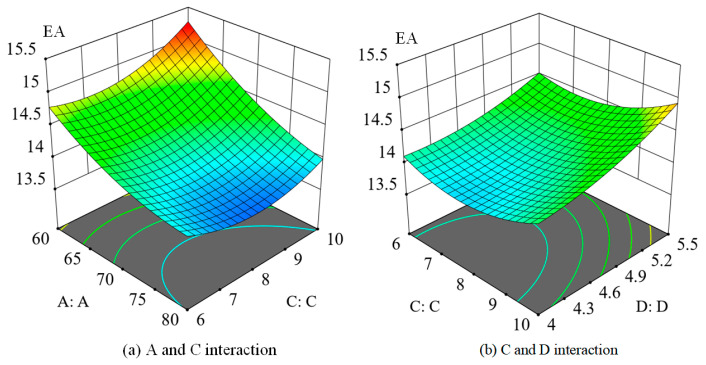
Energy absorption (EA) response surface.

For the variables of radial line length, number of spiral lines, and spiral spacing of the spider web, their impact on the total absorption energy is illustrated in Figure 6. The figure demonstrates that as the radial line length of the spider web increases, the total energy absorption decreases. This is primarily due to the decrease in stress and strain with the increase in radial line length, leading to a reduction in energy absorption. Conversely, an increase in the number of spiral lines and spiral spacing of the spider web tends to increase the total absorbing energy. This is because a higher number of spiral lines and more comprehensive spiral spacing result in an increased distance between the spiral and the center of the spider web. Consequently, this leads to an increased strain on the ring during impact, thereby enhancing the total energy absorption of the spider web.

Furthermore, within the range of 60–80 mm for the radial line length, it can be observed that the radial line length is inversely proportional to the total energy absorbed. In the range of 6–10 turns for the number of spiral lines and 4–5.5 mm for the spiral spacing, the number of spiral lines and spiral spacing are directly proportional to the total energy absorption of the spider webs.

#### 3.4.5. Prediction of the Highest Total Energy Absorption Parameter Combination

**Figure 7:**
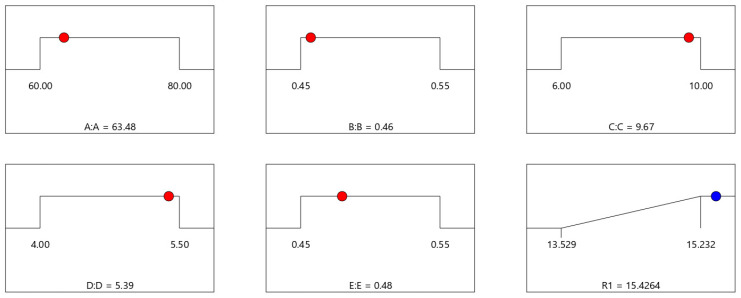
Combination of parameters with the highest total energy absorption.

Parameter combinations with the highest total energy absorption of the spider web obtained from the analysis using Design-Expert software are shown in Figure 7.

From the figure, it can be seen that the total absorption energy is 15.426 mJ at its maximum when the length of the radial line (A) is 63.48 mm, the radial line cross-sectional diameter (B) is 0.46 mm, the number of spiral lines (C) is 10 turns, the spiral spacing (D) is 5.39 mm, and the spiral cross-sectional diameter is 0.48 mm.

## 4. Conclusions

This study begins with a theoretical analysis of the mechanical model of spider webs, identifying several structural factors influencing energy absorption efficiency. Subsequently, the entire process of spider webs intercepting flying insects and absorbing energy is simulated using finite element technology. A simulation analysis is then established using the Box-Behnken Design (BBD) method, with total energy absorption of the spider web as the response and radial line length, radial line cross-section diameter, number of spiral lines, spiral spacing, and spiral cross-section diameter as factors. The response surface method is employed to fit and analyze the relationship between each factor and the response. Based on the summarized energy absorption rules, the following conclusions are drawn:

(1) Spider web radial structure factors have a more significant impact on energy absorption than spiral structure factors. The total absorbed energy of a spider web is inversely proportional to the radial line length of the web.
(2) The number of spiral lines and spiral spacing have negligible effects on the total energy absorption of spider webs. The radial line cross-sectional diameter and spiral cross-sectional diameter have insignificant effects on both complete energy absorption and peak stress.

Through the parameterized study of spider webs, we can gain a more comprehensive understanding of the impact of spider web structure on energy absorption performance, providing insights into broader research on the spider web’s mechanical properties. At the same time, exploring its energy absorption potential can lead to its application in various fields in the future, such as wearable devices, aerospace, and automotive industries. Our investigation focused solely on the structural energy absorption of spider silk, while the energy absorption of spider webs is influenced by other factors, such as material properties and other geometric configurations of spider webs. Therefore, in the future, we will further explore the research on the spider web’s energy absorption by incorporating material and structural considerations.

## Figures and Tables

**Table 1:** Spider web simulation material parameter settings [17].

Material Properties	Numerical Value (after UV Curing)
Density (kg/m^3^)	1080
Young’s modulus (pa)	1.8 × 10^9^
Poisson’s ratio	0.392
Volumetric modulus of elasticity (Pa)	2.78 × 10^9^
Shear modulus (Pa)	6.465 × 10^8^
Yield strength (Pa)	4.8 × 10^7^
Tangential modulus (Pa)	200
Maximum Equivalent Plastic Strain	0.5

**Table 2:** Value range of variables.

Analysis Parameters	A	B	C	D	E
Minimum Value	60 mm	0.45 mm	6	4 mm	0.45 mm
Center Value	70 mm	0.5 mm	8	4.75 mm	0.5 mm
Maximum Value	80 mm	0.55 mm	10	5.5 mm	0.55 mm

**Table 3:** Simulation data sheet.

Experiment Number	A mm	B mm	C Circle	D mm	E mm	EA mJ
1	70	0.5	8	4.75	0.5	14.054
2	60	0.45	8	4.75	0.5	14.809
3	80	0.45	8	4.75	0.5	13.549
4	60	0.55	8	4.75	0.5	15.128
5	80	0.55	8	4.75	0.5	13.749
6	70	0.5	6	4	0.5	14.122
7	70	0.5	10	4	0.5	13.841
8	70	0.5	6	5.5	0.5	14.589
9	70	0.5	10	5.5	0.5	14.607
10	70	0.45	8	4.75	0.45	14.206
11	70	0.55	8	4.75	0.45	14.625
12	70	0.45	8	4.75	0.55	13.762
13	70	0.55	8	4.75	0.55	13.866
14	60	0.5	6	4.75	0.5	14.848
15	80	0.5	6	4.75	0.5	14.394
16	60	0.5	10	4.75	0.5	15.232
17	80	0.5	10	4.75	0.5	14.318
18	70	0.5	8	4	0.45	14.065
19	70	0.5	8	5.5	0.45	14.618
20	70	0.5	8	4	0.55	14.113
21	70	0.5	8	5.5	0.55	14.610
22	70	0.45	6	4.75	0.5	13.979
23	70	0.55	6	4.75	0.5	14.157
24	70	0.45	10	4.75	0.5	14.647
25	70	0.55	10	4.75	0.5	14.285
26	60	0.5	8	4	0.5	14.476
27	80	0.5	8	4	0.5	13.749
28	60	0.5	8	5.5	0.5	15.065
29	80	0.5	8	5.5	0.5	13.804
30	70	0.5	6	4.75	0.45	14.144
31	70	0.5	10	4.75	0.45	14.672
32	70	0.5	6	4.75	0.55	14.168
33	70	0.5	10	4.75	0.55	14.844
34	60	0.5	8	4.75	0.45	14.844
35	80	0.5	8	4.75	0.45	13.577
36	60	0.5	8	4.75	0.55	14.709
37	80	0.5	8	4.75	0.55	13.529
38	70	0.45	8	4	0.5	13.995
39	70	0.55	8	4	0.5	13.948
40	70	0.45	8	5.5	0.5	14.905
41	70	0.55	8	5.5	0.5	14.654

**Table 4:** Total energy absorption significance level test.

Source of Variance	Quadratic Sum	F-Value	*p*-Value	Significance
Models	7.134452234	5.379313901	<0.0001	Highly significant
A	4.41630225	66.59705695	<0.0001	Highly significant
B	0.0196	0.295564534	0.5915	Not significant
C	0.261376563	3.94151234	0.0582	Not significant
D	1.310452563	19.7613929	0.0002	Significant
E	0.08265625	1.246441633	0.2748	Not significant
AB	0.00354025	0.053386344	0.8192	Not significant
AC	0.0529	0.797722645	0.3803	Not significant
AD	0.062001	0.934964116	0.3428	Not significant
AE	0.00189225	0.028534795	0.8672	Not significant
BC	0.0729	1.099319108	0.3044	Not significant
BD	0.010404	0.15689048	0.6954	Not significant
BE	0.02480625	0.374073863	0.5463	Not significant
CD	0.02235025	0.337037818	0.5667	Not significant
CE	0.005476	0.082577112	0.7762	Not significant
DE	0.000784	0.011822581	0.9143	Not significant
A^2^	0.288685227	4.353322176	0.0473	Not significant
B^2^	0.024092742	0.363314295	0.5521	Not significant
C^2^	0.62585347	9.437759646	0.0051	Significant
D^2^	0.14815347	2.234128123	0.1475	Not significant
E^2^	0.031287409	0.471808596	0.4985	Not significant

Where at *p* < 0.001, the factor is highly significant; at *p* < 0.05, the factor is significant; and at *p* ≥ 0.05, the factor is not significant.

## Data Availability

The authors confirm that material supporting the findings of this work is available within the article. The collected data from this work are not available within the article.
